# Multifunctional Carbon-Based Nanocomposite Hydrogels for Wound Healing and Health Management

**DOI:** 10.3390/gels11050345

**Published:** 2025-05-06

**Authors:** Tianyi Lu, Yaqian Chen, Meng Sun, Yuxian Chen, Weilong Tu, Yuxuan Zhou, Xiao Li, Tao Hu

**Affiliations:** 1 School of Mechanical Engineering, Jiangsu Key Laboratory for Design and Manufacturing of Precision Medicine Equipment, Southeast University, Nanjing 211189, China; tylu7@seu.edu.cn (T.L.); sunmeng_seu@163.com (M.S.); 230228092@seu.edu.cn (Y.C.); a18879278919@163.com (W.T.); yx.zhou@seu.edu.cn (Y.Z.); 2 National Engineering Laboratory for Modern Silk, Soochow University, Suzhou 215123, China; 17751637299@163.com; 3 Advanced Ocean Institute of Southeast University, Nantong 226010, China

**Keywords:** carbon-based nanomaterials, multifunctional nanocomposite hydrogels, wound healing, health management

## Abstract

Compared with acute wounds, typical chronic wounds (infection, burn, and diabetic wounds) are susceptible to bacterial infection and hard to heal. As for the complexity of chronic wounds, biocompatible hydrogel dressings can be employed to regulate the microenvironment and accelerate wound healing with their controllable physical and chemical properties. Recently, various nanomaterials have been introduced into hydrogel networks to prepare functional nanocomposite hydrogels. Among them, carbon-based nanomaterials (CBNs) have attracted wide attention in the biomedical field due to their outstanding physicochemical properties. However, comprehensive reviews on the use of CBNs for multifunctional hydrogel wound dressings in the past 10 years are very scarce. This review focuses on the research progress on hydrogel dressings made with typical CBNs. Specifically, a series of CBNs (carbon dots, graphene quantum dots, fullerenes, nanodiamonds, carbon nanotubes, graphene, graphene oxide and reduced graphene oxide) employed in the preparation of hydrogels are described as well as carbon-based nanocomposite hydrogels (CBNHs) with versatility (conductivity, antibacterial, injectable and self-healing, anti-inflammatory and antioxidant properties, substance delivery, stimulus response and real-time monitoring). Moreover, applications of CBNHs in treating different chronic wounds are concretely discussed. This review may provide some new inspirations for the future development of CBNHs in wound care and tissue engineering.

## 1. Introduction

Skin, as the largest respiratory organ (about 2 m^2^) for humans, is made up of three layers: the top epidermis, dermis and deepest hypodermis, which possesses high-performance sensation (touch, pain, and temperature) and remarkable mechanical durability [1]. With such a complex multi-layered tissue structure, human skin serves as a functional boundary to defend against various external invasions, protect the fragile internal environment and maintain the body homeostasis [2,3].

Furthermore, the skin exhibits high anisotropy and viscoelasticity, which are related to the structure of collagen fibers in the dermis layer [4]. The elastic modulus of collagen is 4.4 GPa, significantly greater than the elastic fiber modulus (4.0 MPa) [5]. Owing to the presence of the sweat glands and the porous nature of the stratum corneum, the skin also keeps naturally wet with stable ionic conductivity for a long time [6]. Besides, its temperature can range from 23 °C to 37 °C, varying with the room temperature [7]. A higher skin temperature commonly appears at body parts where the blood vessels are richer and closer to the skin surface [8]. More importantly, skin can play a crucial role in temperature regulation, such as cooling the body via sweating and water evaporation.

However, the exposed skin is highly vulnerable to common injuries from diseases and surgery, and the formed wounds can weaken inherent properties of the skin. The process of skin wound healing is divided into four continuous and coordinated stages, including hemostasis, inflammation, proliferation and remodeling [9]. Specifically, the process of acute or ordinary wound healing is quick and sequential, not requiring extra support, but there are some external factors that hinder the wound healing process, such as infection [10,11], burns [12,13], diabetes [14,15,16], etc. Infected, burn, and diabetic wounds are typically chronic wounds with different complex microenvironments, which are more difficult to heal than incisional and excisional wounds [17]. Compared with infected wounds, burn wounds are accompanied by excessive inflammation and severe infections, while diabetic wounds show persistent hyperglycemia and inflammation, which are more vulnerable to bacterial infections. Accordingly, the antibacterial treatment of chronic wounds is primary and targeted wound management is indispensable.

Although plentiful wound dressings are employed to effectively cover wounds for healing, the dissimilarity of the material compositions and properties in existing dressings and biological tissues largely restrict the combination of dressings and wounds. For instance, traditional dressings such as cotton wool and gauze have been widely used to clean wounds and prevent bacterial infection, but such dry dressings are difficult to ensure the moisture of the wound. Moreover, they adhere to the wound easily, causing damage to the new tissue during their replacement. Consequently, the ideal medical wound dressings with biocompatibility should not only efficiently absorb wound exudate, but also keep the wound site moist and be breathable for easy replacement [18]. Currently, modern wound dressings are greatly improved, especially in their low toxicity, softness, adhesion, moist and mechanical properties. Several wet dressings for clinical application mainly include hydrocolloids, alginates, foams, films and hydrogels [19]. They have high absorbent properties and can maintain a moist environment around wounds. However, hydrocolloid dressings consisting of a self-adhesive hydrophilic colloid granule layer and a waterproof polyurethane (PU) layer cannot absorb high levels of exudate and must be changed many times [20,21]. Alginates without adhesion need to absorb enough fluid to form the gel, and excess fibers may be left in the wounds to trigger inflammatory mechanisms [20,22,23]. As for foams with a porous structure, they can be kept in the wound sites without frequent changes, but the new tissue may be damaged when changing the dressing [24,25]. Film dressings are thin, flexible, and semipermeable, but accumulated exudates may break the seal to the external environment and facilitate the proliferation of bacteria [26]. In contrast, biocompatible hydrogels with a three-dimensional porous structure can absorb a large amount of liquid and maintain their swollen state, which have attracted wide attention from researchers [27].

Flexible hydrogel dressings can help the wounded skin easily adapt to body movements by controllable and low mechanical deformation, and provide the ideal growth microenvironment for cell proliferation, adhesion and migration to accelerate wound healing [28,29]. In recent years, hydrogels have been widely used in biomedical fields [30,31,32], including wound healing, controlled drug release, tissue regeneration, etc. They are prepared through covalent or noncovalent crosslinking of natural or synthetic polymers (e.g., chitosan (CS), gelatin, hyaluronic acid), exhibiting excellent mechanical, electrical, moist, soft, and sensory properties akin to human skin.

Meanwhile, with the booming development of nanomaterials research, new composite hydrogels are being prepared via introducing nanomaterials with unique properties into their networks, realizing the transformation of hydrogels from single function to multi-function and even intelligence [17]. Carbon-based nanomaterials (CBNs), possessing excellent electrical, magnetic, optical, thermal, mechanical and chemical properties [33], not only help to promote various processes of wound healing, including hemostasis, inflammation, proliferation, fibroblast migration, angiogenesis and adhesion, but also contribute to wound care, such as tissue repair, scarless healing and prevention of the loss of tissue integrity [34]. Moreover, CBNs can inhibit bacteria owing to the production of reactive oxygen species (ROS) and their hydrophobicity, which may also act as an antibacterial agent with a delivery property to participate in the four stages of wound healing [35]. With the introduction of CBNs, functionalized hydrogels can be endowed with outstanding mechanical performance, enhanced conductivity, drug delivery and photothermal antibacterial properties. Nevertheless, a comprehensive review of the applications of CBNs in functional hydrogel wound dressings has not been reported.

**Figure 1:**
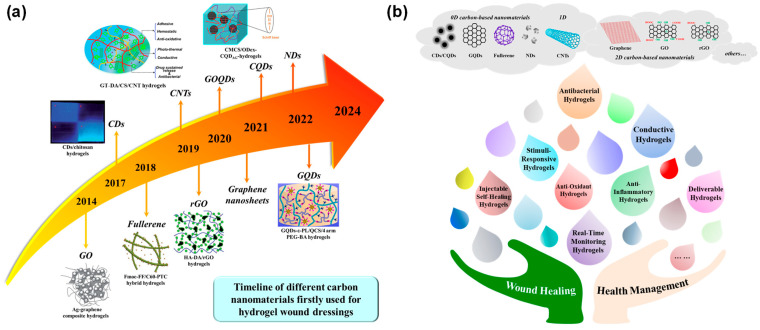
Multifunctional carbon-based hydrogel dressings. (**a**) The timeline of the first fabrication of hydrogel wound dressings using different carbon-based nanomaterials. (**b**) Illustration of versatile carbon-based nanocomposite hydrogels.

So far, original hydrogel wound dressings based on different carbon nanomaterials have been continuously developed, as shown in Figure 1a. This review comprehensively provides an overview of recent achievements, chiefly focusing on the construction of functional hydrogel wound dressings based on the typical CBNs with multiple dimensions. Obviously, there are advanced functions of carbon-based hydrogel dressings, such as conductivity, antibacterial activity, injectable and self-healing properties, anti-inflammatory and anti-oxidation, substance delivery, stimulus response and real-time monitoring (Figure 1b). We have mainly selected the literature on carbon-based nanocomposite hydrogels (CBNHs) used for wound healing and monitoring in the past 10 years from 2014 to 2024, and will concretely discuss multifunctional CBNHs using carbon dots (CDs), graphene quantum dots (GQDs), fullerenes, nanodiamonds (NDs), carbon nanotubes (CNTs), graphene, graphene oxide (GO) and reduced graphene oxide (rGO) and their applications in the treatment and management of different chronic wounds (e.g., infection, burn, and diabetic wounds). Furthermore, the future development trends and prospects of CBNs in wound healing and skin tissue engineering are also discussed.

## 2. Low-Dimensional Carbon-Based Nanomaterials for Multifunctional Hydrogels

Carbon is one of the most plentiful elements on the Earth. Carbon atoms are arranged and combined in various ways and different carbon allotropes are formed, bringing the generation of multifarious CBNs. Due to their great potential in the healing and treatment of diseased and damaged tissues, CBNs have been widely applied in the biomedical field, including the manufacture of hydrogel wound dressings [36]. Common multidimensional CBNs used for hydrogel dressings can be classified into 0D-CBNs (e.g., CDs, GQDs, fullerenes, NDs), 1D-CBNs (e.g., CNTs), 2D-CBNs (e.g., graphene, GO, rGO, etc.), and other carbon nanostructures. In addition, CDs and GQDs have mixed sp^2^- and sp^3^-hybridized carbon along with defects and heteroatoms, while fullerenes and NDs are mainly composed of sp^2^- and sp^3^-hybridized carbon atoms, respectively; CNTs and graphene with its derivatives are mostly made from sp^2^-hybridized carbon atoms [35,37].

As for CBNs with similar orbital hybridization of carbon atoms, their physicochemical characteristics may be dependent on their dimensionalities. With the smallest size, 0D-CBNs exhibit stable fluorescence (e.g., CDs, GQDs) and photosensitive properties (e.g., CDs, fullerenes). They can obtain enhanced antibacterial performance by modification, and serve as nanocarriers (e.g., CDs, NDs). In comparison, 1D-CNTs possess extremely high length-to-diameter ratios, showing splendid mechanical strength and electrical performance. The CNTs are introduced into hydrogels to endow them with conductivity and photothermal antibacterial activity, but their dispersion and modification have always been a problem. Moreover, 2D-graphene with a high specific surface area has eminent optical, electrical, and chemical properties. And GO, as one of its derivatives, is covered with plenty of carboxyls, hydroxyls and epoxides, resulting in extraordinary dispersibility, antibacterial, photothermal and mechanical properties. Compared with CNTs, GO is easy to modify and can carry other substances to achieve synergistic therapeutic effects. rGO is the reduced form of GO, which has superior thermal and electrical properties similar to graphene. Overall, CBNs with different dimensions can be employed for the production of diverse hydrogel dressings, and some significant differences may also exist for the properties of hydrogels prepared with different CBNs of a particular dimensionality. Therefore, in this section, the recent functional advances in carbon-based hydrogel wound dressings are discussed.

### 2.1. 0D-CBNs

#### 2.1.1. Carbon Dots and Carbon Quantum Dots

CDs are surface-functionalized carbon nanoparticles with a small size of less than 10 nm [38]. As a new generation of CBNs, they are easily prepared from carbon nuclei and consist of amorphous or crystalline domains with sp^2^- or sp^3^-hybridized dominance [39,40], which possess high stability, strong hydrophilicity, good biocompatibility, and stable fluorescence properties [41,42,43]. They are also controllable in structure and can act as antibacterial agents and photosensitizers for biomedical applications [44,45]. Meanwhile, carbon quantum dots (CQDs), which actually belong to CDs, are graphene-like crystalline nanospheres with a size of ~10 nm and can exhibit quantum confinement [38]. There are various chemical groups within CQDs that characterize themselves with unique photoluminescence, high chemical stability and photostability, and low cytotoxicity [46,47]. Up to now, CDs have been frequently used for drug delivery [48,49], photodynamic therapy (PDT) [50,51], photothermal therapy (PTT) [52,53,54], etc. And CDs-based nanocomposite hydrogels can effectively integrate the properties of CDs and polymers to exploit their advantages for applications in tissue engineering and health management [55,56,57].

Bacterial infections are a tough challenge in wound healing and may cause a continuous inflammatory response and further delay the healing process [58]. And CDs have been selected as antibacterial agents to effectively destroy bacterial biofilms and inhibit the growth of pelagic bacteria, which obviously exhibit the advantages of durability and environmental protection compared with metal-containing nanocides and traditional antibiotics [59,60,61]. Generally, highly positively charged CDs with a small size are likely to interact with negatively charged bacteria and their cell membranes can be directly disrupted by electrostatic interactions [62]. Cui et al. employed cationic CDs as a significant component of an actual fluorescent antibacterial hydrogel to form physical crosslinks with the polyacrylic acid (PAA) and pectin chains, which played a skeletal adhesion role in the hydrogel network and acted as fluorophores [56]. Importantly, the CDs were further released to kill bacteria and avoid bacterial resistance due to the sensitivity to the changes in the surrounding electrical charge induced by bacterial cells. But Mou et al. revealed the remarkable antibacterial activity of negatively charged CDs [63]. They conjugated anionic CD31 with ɛ-polylysine to engineer a broad-spectrum antibacterial and biocompatible CD-Plys hydrogel with good self-healing and injectability, which accelerated wound healing dramatically.

**Figure 2:**
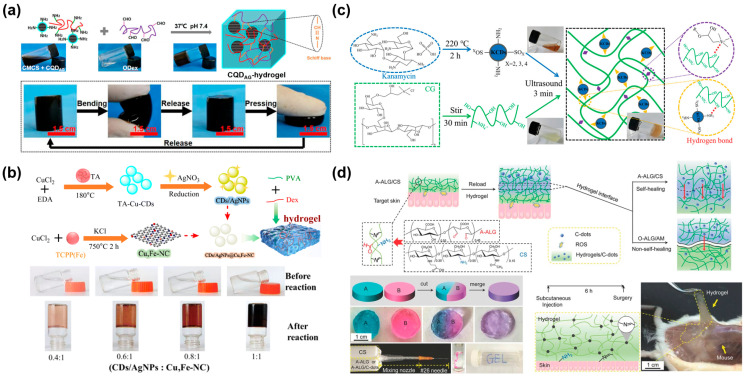
CD/CQD-based hydrogel wound dressings. (**a**) Low-drug resistance injectable self-healing CMCS/ODex-CQD_AG_-hydrogel with potent antibiofilm properties and cutaneous wound healing [55]. (**b**) CG-KCDs hydrogels using kanamycin-derived CDs with antibiotic and photodynamic activities for ROS-enhanced anti-infection effects [65]. (**c**) Adhesive CDs/AgNPs@Cu,Fe-NC hydrogels with antioxidant and anti-inflammatory effects for bacteria-infected wound healing [68]. (**d**) An injectable, biocompatible, and bioadhesive self-healing A-ALG/CS hydrogel with dynamic removal of remote ROS species by reloading the hydrogel’s encapsulating C-dots with intergel diffusive transport [72].

Recent studies have demonstrated that CDs surface-modified with other types of molecules may be loaded into different hydrogel systems to help build multiple properties and improve their antibacterial performance [44,55,64]. For example, Yang et al. used ε-poly(L-lysine) carbon dot (PL-CD) and oxidized dextran (ODA) to fabricate an injectable and self-healing PL-CD@ODA hydrogel based on Schiff bases, which possessed intrinsic antibacterial activity and released PL-CD through a reversible imine bond to achieve a bactericidal effect [44]. Li et al. designed an injectable self-healing hydrogel wound dressing decorated with 2 mg/mL antibacterial carbon quantum dots (CQD_AG_) via the Schiff base linkage between carboxymethyl chitosan (CMCS) and oxidized dextran (ODex), which released CQD_AG_ faster under acidic conditions without drug resistance (Figure 2a) [55]. Sharma et al. prepared a protease-responsive hydrogel to improve angiogenesis and completely restore the epithelium with the sustained release of antibacterial curcumin-derived carbon dots (CurCD) at the wound site [64]. Furthermore, CDs with abundant functional groups (e.g., carboxyl, hydroxyl, etc.) can be doped with one or several metal nanomaterials (e.g., Ag, Cu, Fe, etc.) to enhance the antibacterial properties of hydrogel dressings and promote wound healing [65,66,67]. Zhu et al. synthesized a nanozyme composite and developed a novel mussel-inspired nanozyme hydrogel with high oxidase (OXD)-like activity (Figure 2b) [65]. These nanozyme hydrogels exhibited excellent healing effects on infected wounds and anti-inflammatory activity due to the action of TA (tannic acid)-Cu-CDs reduced-Ag nanoparticles (CDs/AgNPs). Li et al. constructed a Fe-CDs-hydrogel system by encapsulating Fe-CDs nanozymes in an injectable hydrogel to arouse a synergistic antibacterial effect against *E. coli*, *S. aureus*, and methicillin-resistant *Staphylococcus aureus* (MRSA), which promoted collagen deposition and blood vessel formation [66]. Owing to the existence of Fe-CDs with superior biocompatibility and biosafety, the water solubility of hemin was increased considerably.

Moreover, CDs have also been attractive for their unique photosensitive activity, photothermal effects, and material advantages in PDT, which is a promising strategy that produces ROS (e.g., O_2_•, H_2_O_2_, •OH, NO, etc.) against bacterial and biofilm infections [67,68,69,70]. For example, Wang et al. used Cu, N-doped carbon dots (Cu, N-CDs) to prepare a multifunctional Cu, N-CDs@GO-CS hydrogel with strong near-infrared (NIR) absorption for the synergistic treatment of bacteria-infected wounds [67]. The CS hydrogel as a nanocarrier presented an intrinsic antibacterial nature. Beyond that, Cu, N-CDs@GO NCs (nanocomposites, NCs) provided photothermal effects to produce hyperthermia and Cu, N-CDs exerted photodynamic effects to generate ROS. Likewise, kanamycin-sulfate-derived carbon nanodots (KCDs) can be assembled with cationic guar gum (CG) to form a self-healing and injectable hydrogel (CG-KCDs) through noncovalent forces (Figure 2c) [68]. And the CG-KCD hydrogel significantly inhibited the growth of *S. aureus* and *E. coli* with the generation of ^1^O_2_ and •OH by photoexcited KCDs to further reduce apoptotic cells and inflammatory infiltration at the wound sites. Nayak et al. fabricated a pH-responsive and self-healing DNA-CD-PVP (poly(vinylpyrrolidone), PVP) hybrid hydrogel with shape memory by conjugating DNA and PVP polymer to a single carbon quantum dot (CD) simultaneously, which acted as the common nucleus and generated ROS to enhance antimicrobial activity upon visible light irradiation [69]. Especially, a novel hydrogel was designed with integrated functions such as hemostasis, scavenging ROS by an infection-responsive release of tanshinol (Ts) and promoting refractory wound healing. The photothermal effect of CQDs endowed the superoxide dismutase (SOD) mimicking centers with the ability of eradicating infection [70]. However, excessive ROS are probably expressed in the wound, which may trigger strong inflammatory reactions, causing the destruction of cells and impede wound healing by a series of chain reactions including lipid peroxidation, protein denaturation, and DNA damage [71]. Surprisingly, CDs can serve as an antioxidant to inhibit local ROS generation. Chen et al. created a reloadable A-ALG (sodium alginate dialdehyde)/CS hydrogel, which enabled diffusive transport of CQDs across the hydrogel interface to remove remote ROS efficiently and dynamically during the self-healing process (Figure 2d) [72]. The diffusion in the hydrogel was visible and monitored in real time because of the fluorescence of CQDs.

#### 2.1.2. Fullerene

**Figure 3:**
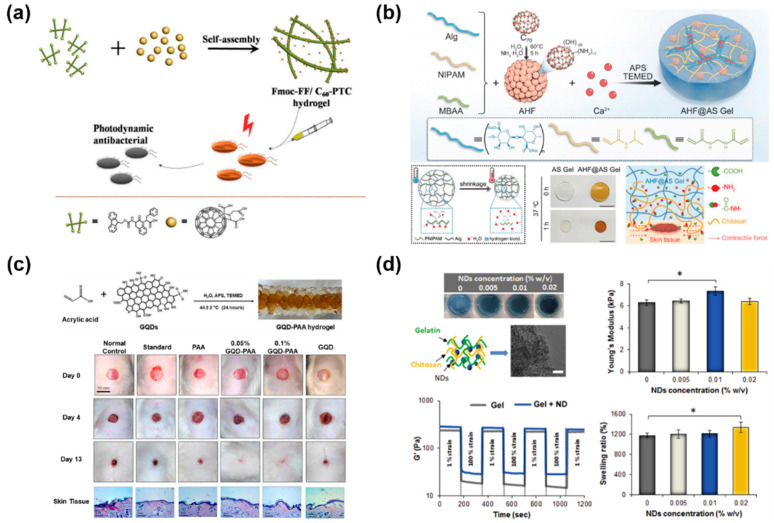
Other 0D-CBNs-based hydrogel wound dressings. (**a**) Injectable Fmoc-FF/C60-PTC hybrid hydrogels for photodynamic antibacterial therapy [78]. (**b**) An antioxidative and active-shrinkage hydrogel (AHF@AS Gel) promoting re-epithelization and skin constriction for wound closure [81]. (**c**) GQD-polyacrylic acid hybrid hydrogel for diabetic wound healing [88]. (**d**) Nanodiamond-based injectable hydrogel for sustained growth factor release [99]. The results are reported as mean ± dev.st (n = 5) * = *p* < 0.05.

The pristine fullerene molecule (C_60_) is comprised of 60 sp^2^ carbon atoms, which are arranged in 20 hexagons and 12 pentagons to form symmetrically a closed spherical structure [73]. Similarly, fullerenes show great potential in efficient PDT due to the sustained production of ROS under white-light irradiation [74,75,76]. And their extended π-conjugated structures and excellent chemical stabilities make fullerenes attractive and different from other photosensitizers [77]. As shown in Figure 3a, a self-assembled hybrid hydrogel combining an amphiphilic small peptide (N-fluorenylmethoxycarbonyl diphenylalanine, Fmoc-FF) with a fullerene derivative (C_60_ pyrrolidine Tris-acid, C_60_-PTC) was designed as an effective photodynamic agent for targeted antibacterial therapy in vivo [78]. Inside the Fmoc-FF/C_60_-PTC hydrogel, the non-covalent interactions between peptides and fullerenes largely inhibited the aggregation of fullerenes.

As nonpolar molecules, fullerenes are hard to disperse in water due to their low solubility. However, they can be chemically modified with hydrophilic groups or macromolecules to improve their aqueous solubility according to recent studies. Chen et al. employed photo-crosslinking to prepare a ROS-scavenging hybrid hydrogel consisting of gelatin methacryloyl (GelMA) and polydopamine (PDA)-coated fullerene (C60@PDA) for wound repair, which accelerated wound closure by 42.9% on day 7 over the control [79]. Kong et al. loaded melanin-glycine-C60 nanoparticles (MGC NPs) into a CS hydrogel to build a two-layer hydrogel system with precise PDT and PTT efficiency in different regions [80]. Under NIR laser (808 nm) irradiation, high-dose MGC NPs offered high ROS/heat for efficient anti-bacterial effects in the upper layer, and the lower layer hydrogel released low-dose ROS with mild thermal performance, which ultimately healed biofilm-infected wounds synergistically. Besides, fullerenes can also obtain antioxidant activity by modification. In Figure 3b, Sun et al. embedded the antioxidative amino- and hydroxyl-modified C_70_ fullerene (AHF) in a thermosensitive active shrinkage hydrogel (AS Gel) composed of N-isopropyl acrylamide (NIPAM) and sodium alginate (Alg), which constructed the AHF@AS Gel to accelerate re-epithelization in both acute and diabetic chronic wounds [81]. Notably, AHF could regulate the state of overexpression of ROS to effectively relieve overactivated inflammation, avoid cellular apoptosis and facilitate fibroblast migration.

#### 2.1.3. Graphene Quantum Dots

GQDs are smaller than 20 nm in size and formed by one to three layers of graphene, which have been referred to as graphene nanofragments [38,82]. The anisotropic GQDs, surface-covered with functional groups, are stable, biocompatible, and easy to modify [83,84,85]. They are similar to CDs and possess high-intensity fluorescence and peroxidase-like activity [35]. Recently, GQDs have shown potential as antibacterial agents due to their outstanding redox properties and Gaussian surfaces, but with low toxicity [86,87]. For example, Shivam et al. investigated the preparation of GQD-PAA hybrid hydrogel and 0.05% and 0.1% GQD-PAA hydrogels accelerated diabetic wound healing, where the agminated antibacterial GQDs exhibited the function of cell signaling (Figure 3c) [88]. In another wound healing system, GQDs were innovatively integrated into bovine serum albumin (BSA)-assisted poly(vinyl alcohol) (PVA) to create PVA/BSA@GQD nanocomposite hydrogels with antibacterial efficacy, which further exhibited a 100% wound closure on Drosophila within 4 h and complete re-epithelialization of mice within 13 days [89]. Besides, GQDs can inhibit bacterial growth with their photothermal effects under visible or near-infrared light. Cheng et al. designed ε-poly-L-lysine grafted graphene quantum dots (GQDs-ε-PL) and prepared a sprayable bacterial responsive hydrogel with a xenon light (400–1100 nm) responsive photothermal performance for the treatment of diabetic ulcers (DUs) [85]. The introduction of GQDs-ε-PL not only improved the mechanical properties of the hydrogel, but also achieved chemo-photothermal synergistic anti-infection capability.

#### 2.1.4. Nanodiamonds

NDs are sp^3^ carbon nanoparticles that generally range from 2 to 10 nm in diameter, which consist of a sp^3^-hybridized carbon core and a sp^2^-hybridized carbon shell [35,37,90]. The surface layer of NDs is coated with oxygen-containing functional groups for stabilization by reducing dangling bonds [91,92,93]. They can be produced using top-down methods like jet milling or abrasion of microdiamonds and detonation of carbon-containing explosives [94,95]. NDs are hard but not dispersible, and possess a high surface area-to-volume ratio with tunable surface structures, which have been explored in coatings [96,97]. Furthermore, NDs have the potential to be introduced into polymeric networks to modulate the properties of hydrogels as nanocarriers [98,99].

Pacelli et al. facilitated an injectable and thermosensitive nanocomposite hydrogel based on gelatin, chitosan and NDs (Figure 3d) [99]. NDs complexed with exogenous human vascular endothelial growth factor (VEGF) were embedded in the hydrogel network, improving its mechanical properties and providing the sustained release of VEGF for wound healing. In another work, NDs were surface-modified precisely under the control of a kinetics model of PDA deposition and an optimized antibacterial PDA-PAM (polyacrylamide, PAM) hydrogel containing NDs-PDA/Ag was further prepared by the regulation of formed AgNPs [100].

### 2.2. 1D-CBNs

CNTs, commonly known as buckytubes, are typically 1D CBNs, which are nanoscale hollow tubes mostly formed from sp^2^ carbon atoms. CNTs have good tensile strength and conductivity, whose aspect ratios (i.e., length-to-diameter ratios) frequently exceed 10,000 [37]. Furthermore, a single graphitic layer or multiple concentric layers can be rolled up into single-walled carbon nanotubes (SWCNTs) or multi-walled carbon nanotubes (MWCNTs), respectively [101]. Double-walled carbon nanotubes (DWCNTs) are considered as another form of CNTs, which are made up of two concentric graphene cylinders [102]. Recently, CNT-based materials have been explored for biomedical applications, such as drug delivery, photodynamic therapies, biosensing, implantable devices, and wound dressing [34]. There is a unique interaction pattern existing between CNTs and biomolecules or cells or even native tissues, such as promoting cell adhesion and proliferation, which may enhance the biological activity of wound dressings [103,104].

CNTs, small and light, present excellent mechanical, stable photothermal and electronic performance, which have been combined with functional hydrogels to enhance their conductivity, mechanical performance, antibacterial treatment, cell adhesion and proliferation, and wound healing [105,106,107,108]. Wang et al. developed a newly regenerated bacterial rBC/PPy/CNT (regenerated bacterial cellulose/polypyrrole/CNT) electroactive hydrogel to enhance cell proliferation with electric fields for wound healing [106]. And Liu et al. incorporated conductive MWCNTs into an elastic GelMA-TA hydrogel to form a wearable strain-sensitive E-skin, which was capable of sensing body motion [107]. Chao et al. reported a LPC (lignin/PVA/CS)-MWCNT composite hydrogel with antioxidant activity and enhanced mechanical properties, which almost completely healed wounds in 21 days under NIR [108]. The CNTs endowed the hydrogel with photothermal antibacterial activity, killing more than 97% of either *E. coli* or *S. aureus* within 5 min and avoiding bacterial resistance.

**Figure 4:**
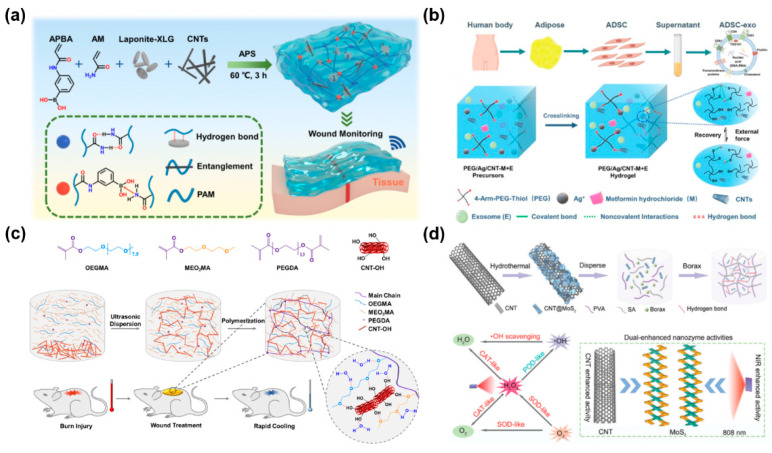
CNT-based hydrogel wound dressings. (**a**) Fabrication of conductive P(AM-APBA)XLG/CNTs hydrogels with robust elasticity and multifunctional responsiveness for flexible sensing and wound monitoring [116]. (**b**) Exosome/metformin-loaded self-healing conductive PEG/Ag/CNT-M + E hydrogel for rescuing microvascular dysfunction and promoting chronic diabetic wound healing by inhibiting mitochondrial fission [121]. (**c**) PMO-CNT hydrogels with improved cooling performance via integrating thermal conductivity and heat storage capacity for burn therapy [122]. (**d**) Adaptive hydrogels (PSCMo) based on nanozyme with dual-enhanced triple enzyme-like activities for wound disinfection and mimicking antioxidant defense system [129].

However, the aggregation of CNTs is common in a complex system, which can inevitably cause problems such as poor conductivity and inhomogeneous mechanical properties [109,110,111]. As a solution, chemical treatment may disrupt π-conjugation of CNTs and thereby change their electronic properties [112]. In contrast, noncovalent modification is more acceptable, involving physical adsorption of functional moieties on the CNT surface, such as π–π stacking, hydrophobic, electrostatic and van der Waals forces [113]. For example, a series of conductive self-healing and adhesive nanocomposite hydrogels, with photothermal antibacterial properties and pH-responsive release capacity, were developed based on N-carboxyethyl chitosan (CEC) and benzaldehyde-terminated pluronic F127/carbon nanotubes (PF127/CNT) [114]. The addition of CNTs endowed the hydrogels with good conductivity (e.g., 8.45 × 10^−3^ S m^−1^ for CEC/PF/CNT4 hydrogels) and photothermal antimicrobial activity. Their self-healing and mechanical properties were also improved owing to strong π–π stacking and hydrophobic interactions among individual CNTs. Li et al. proposed SWCNT-based hydrogel composites exhibiting bulk conductivity (1.27 S m^−1^ with 8 mg mL^−1^ SWCNTs) with a fast and autonomous self-healing ability that restored 95% of the original conductivity within 10 s under ambient conditions [115]. Essentially, pyrene moieties facilitated the dispersion of SWCNTs due to their strong interaction with the sidewalls of the CNTs through π–π stacking. In Figure 4a, 3-acrylamidophenylboronic acid (APBA), acrylamide (AM), and LAPONITE^®^ XLG nanosheet (XLG) stabilized CNTs were employed to prepare a hydrogel-based E-skin (P(AM-APBA)XLG/CNTs) with robust elasticity and multifunctional responsiveness for flexible sensing and wound monitoring [116]. The embedded CNTs were well entangled with molecular chains to enhance their mechanical properties and they formed a conductive network to improve the conductivity and sensing properties of the hydrogel. Xiao et al. incorporated cellulose nanocrystal grafted phenylboronic acid (CNCs-ABA) and MWCNTs into PVA to design a fast healable and shape memory ECH with excellent biocompatibility [117]. The MWCNTs were well-dispersed and stabilized by nanocellulose by virtue of their electrostatic repulsion. In another work, Xu et al. developed antimicrobial macroporous nanocomposite hydrogels (MNHs) for neural stem cell differentiation and infected wound healing. They were generated from an air-in-water emulsion template stabilized by colloidal hybrids of CNTs and GelMA [118]. Specifically, the GelMA served as a surfactant and exhibited good binding ability with the colloidal particles, improving the dispersion of CNTs through its physical absorption effect. Moreover, the MNH hydrogels demonstrated a tunable pore size, electrical conductivity and mechanical properties with various CNT concentrations in the crosslinking matrices.

For CNTs, biomolecules or other nanomaterials may be coupled to their functional ends such as hydroxyl and carboxyl groups, which are generated by oxidization in strong acid [119]. And their tubular and large surface area can support the adsorption and/or conjugation of various therapeutic drugs for disease treatment [120]. Based on this, Forero-Doria et al. prepared supramolecular hydrogels of cellulose with sustained release of therapeutic substances for wound healing [119]. The MWCNTs were conjugated to increase the loading capacity of bioactive compounds, such as allantoin, dexpanthenol, resveratrol and linezolid. Zhang et al. engineered exosome/metformin-loaded hydrogels with self-healing and conductive properties, which can rescue microvascular dysfunction and accelerate chronic diabetic wound repair via inhibiting mitochondrial fission (Figure 4b) [121]. Hydroxyl-modified MWCNTs with good conductivity were incorporated into the hydrogels to form hydrogen bonds with thiol, finally yielding a stable 3D structure. Another novel hydrogel dressing for burn wound treatment was easily prepared by in situ cross-linking polymerization of poly(ethylene glycol) (PEG) dimethacrylate (PEGDA), 2-(2-methoxyethoxy) ethyl methacrylate (MEO_2_MA) and oligo(ethylene glycol) methacrylate (OEGMA) via a one-pot method. The thermal conductivity was increased via blending and dispersing hydroxylated multiwall CNTs (CNT-OH) into the hydrogel (Figure 4c) [122]. The heat storage capacity and thermal conductivity was integrated to improve its cooling efficiency and reduce heat damage.

Furthermore, in view of the strong hydrophobic interaction between individual CNTs, surface coating has been used to efficiently improve the dispersion of CNTs and reduce their cytotoxicity [123,124]. Liang et al. employed gelatin-grafted-dopamine (GT-DA) and polydopamine-coated carbon nanotubes (CNT-PDA) to engineer antibacterial, adhesive, antioxidant and conductive GT-DA/chitosan/CNT composite hydrogels through the oxidative coupling of catechol groups using a H_2_O_2_/HRP (horseradish peroxidase) catalytic system [125]. CNT-PDA provided these hydrogels with excellent photothermal antibacterial activities against Gram-positive and Gram-negative bacteria. Kittana et al. showed that SWCNTs and MWCNTs were complexed with chitosan via van der Waals’ interactions and the chitosan polymer wrapped on to the CNT with its hydrophilic regions, producing a hydrogel incorporating a scaffold of CNTs [126]. And Cirillo et al. used gelatin-coated MWCNTs as an electro-conductive component to synthesize electro-responsive hybrid hydrogels by free radical polymerization [127]. Lin et al. synthesized multifunctional hydrogels for combined treatments of bacterial diabetic wounds, which were formed by cross-linking of oxidized dextran (ODex) and caffeic acid grafted chitosan (CACS) with the introduction of a metal-coordinated tubular nanocomplex OCNT@COF-Fe (O@CF, covalent organic framework, COF) [128]. Besides, O@CF, containing a uniform COF-coated oxidized carbon nanotube (OCNT), exhibited synergistic photothermal-combined multiple photodynamic properties for sterilizing and hypoxia relief as an augmented photosensitizer. In another study, molybdenum disulfide nanosheets (MoS_2_ NSs) were loaded onto CNTs and treated with NIR, significantly enhancing the triple enzyme-mimicking activities of MoS_2_ (Figure 4d) [129]. And then the nanozyme was composited into a multifunctional hydrogel in order to eradicate bacteria and eliminate free radicals.

### 2.3. 2D-CBNs

Graphene is a 2D sheet of sp^2^-hybridized carbon atoms organized in a hexagonal lattice with a single or few atomic layers [130], exhibiting outstanding optical, electrical, and chemical properties [131]. Among the available conductive nanomaterials, graphene tends to meet the specific application requirements of great mechanical properties and stable electrical conductivity [132,133], especially in hydrogel dressings [134,135]. Luo et al. used the bio-composites of graphene, MXene sheets, hyperbranched polyglycidyl ether (HBPG), CS, and hemostatic chitosan/human-like collagen (HLC) to form a 3D double-network hydrogel through chemical and physical dual-crosslinking [135]. The hydrogel not only possessed hemostasis, moisture permeability, mechanical flexibility, electroactivity, antibacterial activity and self-healing properties for wound treatment, but also underwent real-time monitoring of large-scale human motion by providing detailed data to guide clinical practice. There are two typical forms of graphene derivatives for hydrogel wound dressings: GO and rGO. GO is a graphene-based material with a hexagonal and crystalline structure with a layer of oxygen on its sheets, which makes GO interact better with functional materials [136]. rGO is the reduced form of GO, which has superior thermal and electrical properties similar to pristine graphene [137,138,139]. Besides, both GO and rGO possess relatively better aqueous solubility than pristine graphene and these graphene-based materials can be modified to obtain biological functionality and attractive optical performance [140,141].

**Figure 5:**
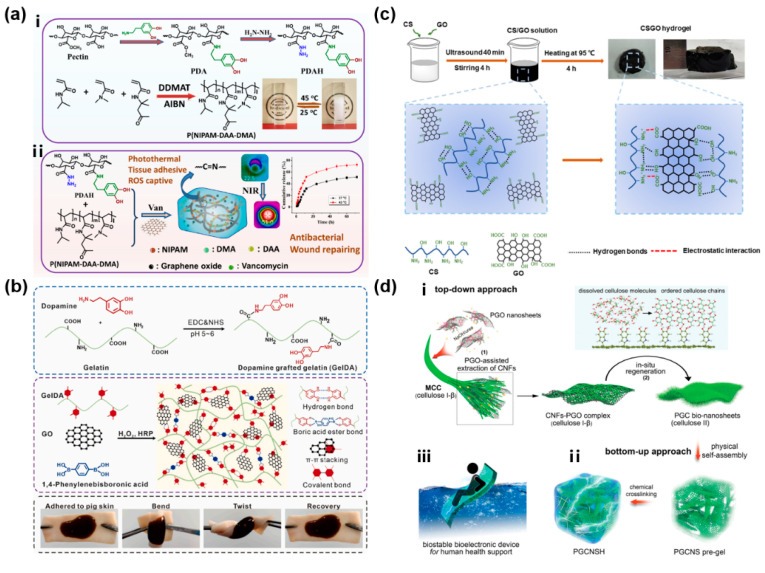
GO-based hydrogel wound dressings. (**a**) Biodegradable pectin-based thermo-responsive composite GO/hydrogel with mussel inspired tissue adhesion for NIR enhanced burn wound healing: (i) the synthesis of PDAH and P(NIPAM-DAA-DMA); (ii) the fabrication of a versatile hydrogel with various functions for wound repair [142]. (**b**) Adhesive GelDA/GO hydrogels with self-healing, hemostasis, and electrical conductivity for electromyography (EMG) monitoring [144]. (**c**) Shear-thinning and self-healing chitosan–graphene oxide hydrogel for hemostasis and wound healing [146]. (**d**) Conductive cellulose bio-nanosheet assembled biostable hydrogel (PGCNSH) for reliable bioelectronics: (i) top-down approach for the formation of PGC bio-nanosheets; (ii) bottom-up approach for the formation of PGC bio-nanosheet-assembled hydrogel (PGCNSH) through physical assembly and chemical crosslinking; (iii) conductive and biostable PGCNSH as a reliable bioelectronic device to support human health in aqueous environments [183].

GO, with plenty of carboxyls, hydroxyls and epoxides, has exhibited extraordinary biocompatibility, dispersibility, amphiphilicity, antimicrobial, thermal and mechanical properties, which are used in the fabrication of hydrogels, mainly for wound healing and management currently [142,143,144,145]. Chen et al. regulated the LCST to body temperature and cross-linked poly (N-isopropyl acrylamide) (PNIPAM) based thermo-responsive copolymers with dopamine functionalized pectin hydrazide (PDAH) in order to fabricate a biodegradable self-healing hydrogel with injectability for NIR enhanced burn wound healing (Figure 5a) [142]. In this research, the added GO took advantage of its photothermal property to enhance its antibacterial activity and the NIR irradiation also accelerated the vancomycin release rate based on photothermal induced phase transition. To disrupt bacterial biofilms and avoid antibiotic resistance, Wang et al. incorporated GO reduced by ascorbic acid and rare earth terbium ions (Tb^3+^) in a PVA-alginate hydrogel for treating infected chronic wounds [143]. The rGO weakened the hydrogen bonding between PVA and alginate and induced loosening of the hydrogel network. Consequently, the increased loading and release of Tb^3+^ resulted in a synergistic antibacterial effect between Tb^3+^ and rGO. For another, Han et al. mixed dopamine grafted gelatin (GelDA) with 1,4-phenylenebisboronic acid and GO to obtain adhesive GelDA/GO hydrogels with self-healing and hemostasis by an H_2_O_2_/HRP catalytic system (Figure 5b) [144]. The addition of GO enhanced the mechanical properties of hydrogels as well as their electrical conductivity. Furthermore, hydroxylated graphene (GOH), another graphene-based nanomaterial, was combined with aminophenylboronic acid grafted sodium alginate (Alg-PBA) and PVA to form the Alg-PBA/PVA/GOH hydrogel with injectability, self-healing, motion monitoring via dynamic interactions and supramolecular interactions [145]. With the assistance of GOH, the hydrogel obtained increased mechanical strength and conductivity, realizing in situ bacterial sensing and killing functions.

During the synthetic process of hydrogels, noncovalent or covalent bonds generally play a crucial role in the direct bonding of GO/rGO sheets to polymers or other nanomaterials [146,147,148,149,150,151,152]. As shown in Figure 5c, Feng et al. reported an adhesive and hemostatic CSGO hydrogel facilely prepared by one-pot heating of a mixture of chitosan (CS) and GO [146]. The dynamic reversible breakage and recombination of noncovalent bonds between CS and GO, which included electrostatic interactions and hydrogen bonds, brought injectability and self-healing abilities to CSGO hydrogels. Another GO-based injectable and self-healing hydrogel dressing (CHGB) was designed for chronic infected diabetic wounds by a dynamic Schiff-base reaction and electrostatic interactions between oxidized hyaluronic acid, N-carboxyethyl chitosan, GO, and polymyxin B [147]. Particularly, the introduction of GO enhanced its mechanical properties and imparted excellent conductivity and immune regulation to the CHGB hydrogel. In another work, a GelAlg@rGO-Pev (gelatin-alginate/rGO/platelet-derived extracellular vesicles) gel with promising macrophage polarization and reactive oxygen species (ROS)-scavenging capability was reported for diabetic wound healing [148]. The incorporation of rGO with similar properties to GO enhanced the mechanical modulus of the hydrogel through polymeric coordination bonding and adjusted its micromorphological structure. And the rGO, with strong NIR absorption ability, acted as an excellent photothermal agent for photothermal-derived hyperthermia to form heat shock proteins (HSP).

Moreover, the existence of various oxidizing functional groups and large specific surface area makes GO easily grafted or functionalized in order to improve the properties of synthesized hydrogels. Hydrogels based on different functionalized GO nanomaterials are shown in Table 1.

GO can be modified with various nanomaterials, such as some polymers, metal nanoparticles (NPs), and other organic nanomaterials. Polymers, such as PDA [163,173,175], QN [153], PEI [154], CS [156], BPEI [162], arabinoxylan [176], CMCS [178], ε-poly-L-lysine [181], and BC [184], have been studied due to their excellent biocompatibility and reactive functional groups. Among these polymers, PDA has been widely used to reduce GO, and the GO can be coated by PDA (Figure 5d) [183]. For example, PDA-mediated GO obtains reasonable hydrophilicity, high photothermal efficiency, good conductivity, and rich surface-active sites, which can thereby provide superior antibacterial effects and effective cell adhesion and proliferation for the treatment of chronic wounds [170,179]. Besides, PDA can modify GO simultaneously with some metal nanoparticles like Ag NPs and Fe_3_O_4_ [158,161,185]. The metal nanoparticles used for the modification of GO in the hydrogel network mainly contain metal-doped organic and inorganic metal nanoparticles, which may combine with other nanomaterials and generate synergistic effects. Specifically, they can be combined with metal-doped organic NPs like Cu, N-CDs [67] or with inorganic metal NPs involving Ag-based NPs [157,177,181], MoS_2_ [157], ZnO QDs [166], Fe_3_O_4_ [185], Bi_2_S_3_ and TiO_2_ NPs [187]. As for Ag NPs, commonly endowed with broad-spectrum antibacterial activity and reduced bacterial resistance, they have been incorporated into GO-based hydrogels to enhance the photothermal antibacterial effect and are supported by the GO substrate to reduce their aggregation and improve their dispersibility [158]. And some other metal nanoparticles can also increase their light conversion efficiency and enhance their photoelectric response [187].

## 3. Multifunctional Carbon-Based Nanocomposite Hydrogels for Chronic Wound Healing

There are different types of skin wounds requiring wound dressings with various functions. Generally, sudden external damage to the skin structure may concisely cause the formation of acute wounds like superficial skin injury and elective operation wounds [188]. And with standard wound treatment and care, acute wounds can be healed through a healing routine within 1–2 weeks [189]. However, skin wound healing in practice is extremely complicated and depends on the complex synergistic interaction of highly regulatory factors to restore the skin barrier function [17]. Meanwhile, the wound healing process tends to be affected and disrupted by many factors, such as infection, oxygen supply, chronic diseases, and even genetic factors [36]. Accordingly, chronic wounds refer to wounds with delayed healing more than 3 months, where the wound fluid may inhibit cell proliferation and angiogenesis [190,191,192]. In addition, chronic wounds are vulnerable to outside influences, leading to the prolongation of the inflammatory period and blocking the transition from inflammation to the remodeling period [193,194,195]. In this section, typical chronic wounds, such as infected, burn, and diabetic wounds, are discussed as well as the applications of CBNHs for promoting chronic wound healing.

### 3.1. CBNHs for Infected Wound Healing

The wound healing process is inevitably challenged by bacterial infections, which always cause persistent inflammation at the infected wound sites and even the failure of wound repair. Nowadays, there are types of effective antibacterial strategies widely used in wound healing, such as antibiotics and other antibacterial drugs, inorganic metals and metal oxides, photothermal antibacterial, photodynamic antibacterial, and cationic polymers [17]. However, bacterial resistance has become increasingly serious with the use of antibiotics [196], and metal-based biomaterials for wound dressings are faced with the biotoxicity and potential risks of long-term retention [71]. Accordingly, various antibacterial hydrogels using antibacterial substances have been designed to solve these problems through physical loading or chemical reactions [197,198,199]. Among the available nanomaterials with low toxicity, CBNs with many advantages, including low preparation cost, good mechanical stability, strong biocompatibility, biodegradability and antibacterial activity, have attracted much attention from researchers.

**Figure 6:**
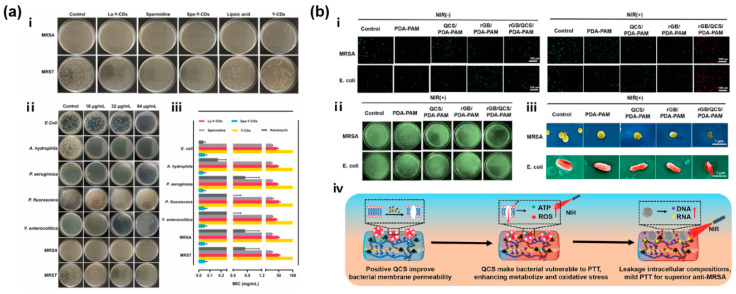
(**a**) Antibacterial properties of SCDs-AP hydrogels: (i) representative colony formation of MRSA and MRST on LB agar plates in the untreated and treated with La-Y-CDs, spermidine, Spe-Y-CDs, lipoic, and Y-CDs (at a concentration of 25 mg/mL); (ii) the MIC of Spe-Y-CDs against seven bacteria; (iii) comparison of MICs of La-Y-CDs, spermidine, kanamycin, Spe-Y-CDs, and Y-CDs against seven bacteria (n = 4, mean ± SD) [56]. (**b**) Antibacterial activity and mechanism of rGB/QCS/PDA-PAM hydrogel: (i) fluorescence images of the live/dead (green/red) bacteria treatment via different hydrogels with or without mild NIR irradiation (0.8 W/cm^2^, 600 s); (ii) optical images of MRSA and *E. coli* bacterial colonies after different treatments under mild NIR irradiation (0.8 W/cm^2^, 600 s); (iii) SEM image of MRSA and *E. coli* showing the changes of MRSA and *E. coli* after treatment with different hydrogels under mild NIR irradiation (0.8 W/cm^2^, 600 s); (iv) antibacterial mechanism of the rGB/QCS/PDA-PAM hydrogel, and the upward red arrow indicates an increase [159].

CBNs with inherent antimicrobial properties can act as antibacterial agents involved in wound healing, owing to the intermolecular interactions or even ROS generation. For example, a fluorescent CDs-releasing SCDs-AP (spermidine-CDs with AA and pectin) hydrogel showed long-term potent broad-spectrum antibacterial ability (Figure 6a) [56]. Among the CDs of three different zeta potentials, the synthesized antibacterial high-cationic Spe-Y-CDs (+51.20 mV) were released from the hydrogel in response to broken hydrogen bonds caused by the growing bacteria, seriously damaging the bacterial membrane. Meanwhile, some CBNs, including CDs, CNTs, GO, rGO and so on, possess photothermal ability for PTT, and some of them, like fullerenes and CDs with unique photosensitive activity, can even function in PDT. Importantly, antibacterial CBNHs have been prepared by combining CBNs with one or several antibacterial nanomaterials, such as metal-based nanoparticles, polymers, and antibacterial drugs, which can not only improve their integrated properties but also achieve a synergistic antibacterial effect. In a CNT@MoS_2_ NSs incorporated PVA/sodium alginate (PSCMo) hydrogel, CNTs loaded with MoS_2_ NSs promoted the nanozyme activities of MoS_2_ significantly through NIR irradiation [129]. According to an antibacterial test in one study, CNT@MoS_2_ nanosheets with 20 wt% CNT were demonstrated to possess the best antibacterial performance due to having the best photothermal conversion ability and peroxidase-like activity.

A versatile hydrogel dressing (rGB/QCS/PDA-PAM) with skin adaptiveness and mild photothermal antibacterial activity was developed to inhibit MRSA infection and accelerate infected dynamic wound healing (Figure 6b) [159]. Firstly, glycocalyx-mimicking phenylboronic acid on 3-aminophenylboronic acid modified rGO (rGB) specifically captured abundant bacteria exposed to the hydrogel, and then QAS decorated carboxymethyl chitosan (QCS) punctured the membrane of the trapped bacteria. Subsequently, the rGB achieved efficient synergistic antibacterial activity through mild PTT. Furthermore, Huang et al. prepared a PDA@Ag5GO1 composite with PDA, Ag and GO, which was then introduced into PNIPAM hydrogel to form an excellent antibacterial and high self-adhesive hydrogel with NIR driven shrinkage [158]. Apparently, no bacterial colonies existed on the agar plate in the PGH2 group, indicating that 2 mg mL^−1^ PDA@Ag5GO1 hydrogel possessed good antibacterial properties. Lu et al. developed a bio-adhesive and antibacterial bandage, using chitosan/graphene oxide (CSGO) hydrogel and a piezoelectric nanogenerator (PENG) patch based on electrospun polyvinylidene difluoride (PVDF) nanofibers, which promoted infected wound healing in 21 days [200]. In addition, Fan et al. prepared Ag-graphene hydrogels using Ag-GO composites, acrylic acid (AA) and N,N′-methylene bisacrylamide (BIS), and the hydrogel with an optimal Ag to graphene mass ratio of 5:1 (Ag5G1) exhibited excellent antibacterial abilities. The Ag5G1 hydrogel also efficiently accelerated wound healing in 15 days [201].

### 3.2. CBNHs for Diabetic Wound Healing

The number of patients suffering from diabetes mellitus is rising worldwide, which seriously endangers their lives and health along with complications such as chronic diabetic wounds, nerve damage, renal failure, eye diseases, and cardiovascular diseases, etc. [202,203,204]. Diabetic wound healing can be related to the physiology and pathology of diabetes [205,206]. Persistent hyperglycemia can lead to the overexpression of pro-inflammatory cytokines, and hinder angiogenesis and re-epithelialization [207,208]. And excessive ROS accumulated in diabetic wounds can inhibit the migration of endogenous stem cells, phagocytes, and macrophages, and induce robust inflammatory reactions to make wounds vulnerable, which also causes lipid peroxidation, protein denaturation, and even DNA damage [209,210,211]. Furthermore, ROS-mediated excessive oxidative stress, sustained inflammation, degradation of extracellular matrix proteins, etc., can lead to nerve cell dysfunction and death [212]. Most importantly, bacterial infection poses a great threat to diabetic patients with low immunity.

**Figure 7:**
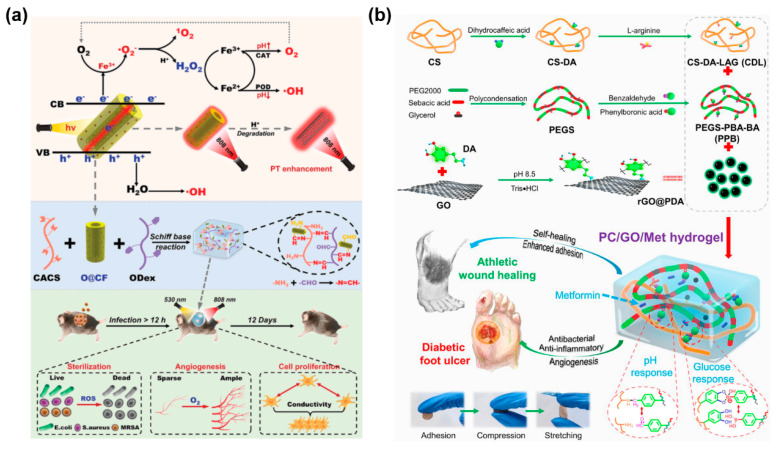
(**a**) O@CF@G hydrogel with ROS/O_2_ generation and photothermal enhancements for accelerating bacterial diabetic wound healing [128]. (**b**) pH/glucose dual responsive metformin release PC/GO/Met hydrogel dressings with adhesion and self-healing via dual-dynamic bonding for athletic diabetic foot wound healing [170].

Owing to the complex physiology and pathology of diabetes, diabetic wounds are generally more fragile than common infected wounds. However, diabetic wound healing rates can be increased by employing CBNHs that satisfy some essential functional requirements, such as cell and cytokines delivery; reducing blood glucose; and exhibiting antibacterial, anti-inflammatory, and angiogenesis properties [213,214,215,216]. Multifunctional CBNHs, comprising enhanced or functionalized CBNs, are designed as nanocarriers and release therapeutic drugs via their uniform porous structure to suppress the bad symptoms of diabetes, accelerating diabetic wound healing. An antimicrobial pH and thermosensitive hydrogel modified with ROS scavenging carbon nanodots was formed to deliver human amniotic membrane derived stem cells (hAMSCs), which led to stimulating early angiogenesis, superior collagen deposition, and complete diabetic wound healing in 21 days [217]. As for exosome/metformin-loaded PEG/Ag/CNT-M+E hydrogels, they triggered cell proliferation and angiogenesis, relieved peritraumatic inflammation and vascular injury, and promoted wound healing. The hydrogels not only reduced the level of ROS via interfering with mitochondrial fission, but also protected F-actin homeostasis and alleviated microvascular dysfunction [121]. As shown in Figure 7a, the O@CF@G hydrogels, armed with Fe-doped COF-coated oxidized CNTs, can well adhere to diabetic wounds for imbibition, hypoxia alleviation, bacteria killing, drug-resistant biofilm elimination, and intercellular electrical signal conduction [128].

Moreover, the adhesive self-healing PC/GO/Met hydrogels possessed stimuli-responsive metformin release ability and easy removability, promoting chronic athletic diabetic wound healing. They also provided a local-specific drug dual-response release strategy for the treatment of type II diabetic feet (Figure 7b) [170]. The addition of metformin (Met) and GO, as well as their synergy, were confirmed to better promote wound repair in vivo. Dou et al. made PAM-based hydrogels using well-reduced and uniformly dispersed Hep (heparin)-PDA-rGO nanosheets, which showed commendable conductivity (3.63 S/m) and sensor performance. The multi-functional conductive hydrogels scavenged ROS, mitigated inflammation, and enhanced angiogenesis in diabetic wounds [164]. He et al. developed photothermal antibacterial antioxidant conductive self-healing hydrogels based on CMCS, 2,3,4-trihydroxybenzaldehyde (THB), copper chloride (CuCl_2_), and GO-N,N′-di-sec-butyl-N,N′-dinitroso-1,4-phenylenediamine (BNN6), which can release nitric oxide and accelerate wound healing in Type I diabetes [172]. Tu et al. designed a thermosensitive conductive polydopamine-modified GO hydrogel (GDFE) with F127-EPL. The in vivo diabetic wound model demonstrated that GDFE can rapidly promote wound healing through fast anti-inflammation and angiogenesis and M2 macrophage polarization [179].

### 3.3. CBNHs for Burn Wound Healing

A burn wound is a form of skin damage caused by thermal, chemical, electrical, or radiation damage [122], mostly accompanied by excessive inflammation, severe infections, reduced angiogenesis, tissue edema and necrosis, which can delay healing, lead to multiple organ failure and even endanger lives [218,219]. Once the skin is directly exposed to high temperature, the fluid may be lost rapidly and dangerously, and the coagulation and loss of proteins can lead to irreversible tissue damage and susceptibility to infection [32,220]. Therefore, plentiful moist antibacterial hydrogel dressings with anti-inflammatory and pro-angiogenic properties have been designed for burn wound healing, which are easy to remove and can deliver therapeutic substances such as drugs, cells, and cytokines [142,151,221].

**Figure 8:**
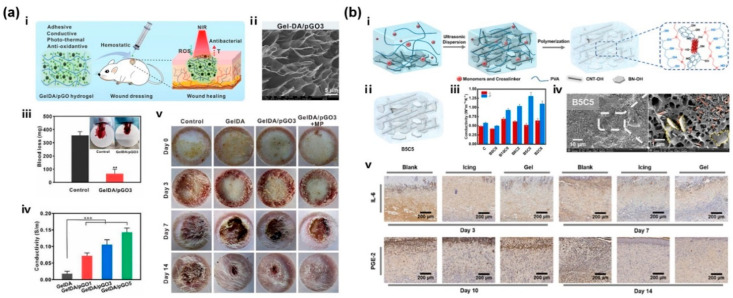
(**a**) A multifunctional mussel-inspired GelDA/pGO hydrogel with antioxidant and electrical conductivity and photothermal activity loaded with mupirocin for burn healing: (i) GelDA/pGO hydrogels with potential application in wound closure; (ii) SEM images of GelDA/pGO3 hydrogels; (iii) blood loss in control group and GelDA/pGO3 treated group, all scale bars indicate means ± standard deviations (n = 3), ** *p* < 0.01; (iv) conductivity of the hydrogels, *** *p* < 0.001; (v) photographs of wounds at 0, 3, 7, and 14 d for PBS, GelDA, GelDA/pGO3 and GelDA/pGO3 + MP hydrogel [171]. (**b**) Enhanced thermal conductive and moisturizing PMO-CNT hydrogels by constructing 3D networks of BN-OH and CNT-OH in alignment for burn therapy: (i) schematic representation of the 3D structure of the hydrogel; (ii) the fillers alignment in the B5C5 hydrogels; (iii) thermal conductivity of the hydrogels; (iv) SEM images of B5C5 hydrogels; (v) IL-6, PGE-2 and VEGF stained immunohistochemical sections [222].

Among them, multifunctional CBNHs with remarkable mechanical properties and a photothermal antibacterial effect have drawn extensive attention. For example, Babaluei et al. developed an injectable silk fibroin/carboxymethyl cellulose/agarose (SF/CMC/AG) hydrogel for full-thickness burn healing, which contained PDA functionalized GO with conductivity, hemostasis, antibacterial, and anti-oxidant properties [167]. According to Figure 8a, a series of adhesive and hemostatic GelDA/pGO (PDA-reduced GO, pGO) hydrogels with antioxidant, electrical conductivity and photothermal antibacterial activity were designed [171]. And with the loading of mupirocin, the burn wound treated by GelDA/pGO3 + MP (Mupirocin, MP) presented the most excellent effect in promoting wound healing. Furthermore, CBNHs can also exhibit great thermal conductivity to reduce heat damage and improve healing efficiency. Shi et al. dispersed CNT-OH in order to form a PMO-CNT (PEGDA/MEO_2_MA/OEGMA, PMO) hydrogel with a thermally conductive network and cooling performance for burn therapy, which consisted of thermally responsive PEG derivatives [122]. The CNT-OH could rapidly absorb heat, further reduce thermal damage and promote wound healing. In Figure 8b, the enhanced thermal conductive and moisturizing hydrogels were prepared by constructing 3D networks of BN-OH and CNT-OH in alignment [222]. The thermal conductivity of the B5C5 hydrogels was enhanced up to 1.31 W m^−1^ K^−1^ and 226% to the pristine hydrogel of 0.58 W m^−1^ K^−1^. And the treated burn wounds expressed less pro-inflammatory IL-6 and the allogenic substance PGE-2 during the late phase of the healing process.

## 4. Carbon-Based Nanocomposite Hydrogels for Real-Time Wound Monitoring and Health Management

The dynamic changes in wounds occurring during the healing process can influence their closure, including pH, glucose level, temperature, surface tension, etc., which need to be monitored in real time [14,223]. Undoubtedly, the pH value of the wound microenvironment is one key factor closely related to wound diagnosis and treatment, affecting many physiological processes, including the inflammatory response, collagen formation, and angiogenesis [224,225]. Generally, with the effect of active neutrophils, acute wounds have a relatively low pH value between 4–6, while chronic wounds are more alkaline at pH 7–9 and susceptible to bacterial infections [226,227]. In addition, the glucose level is another crucial factor for the guidance of clinical treatment, which correlates with diabetic wound status and can serve as a prognostic indicator for diabetes [228,229]. Therefore, monitoring the wound pH and glucose level can help identify infection risks and allow for the adjustment of treatment strategies immediately.

**Figure 9:**
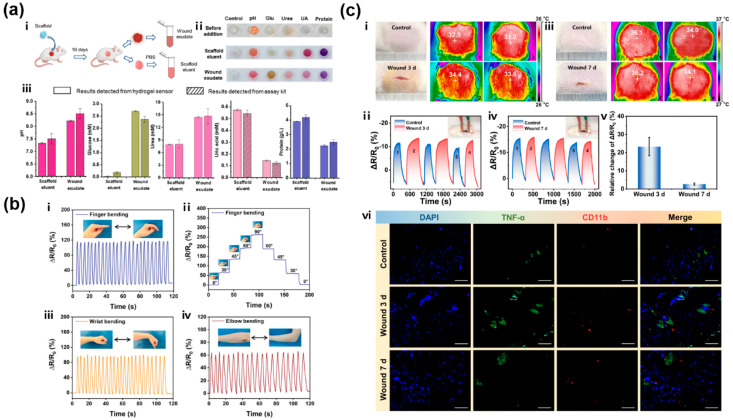
(**a**) A CD-doped hydrogel sensor array for multiplexed colorimetric detection of wound healing: (i) acquisition process of two types of wound fluids from rats (scaffold eluent vs. wound exudate); (ii) color changes of sensors from left to right, control, pH, glucose, urea, uric acid (UA) and protein before and after adding wound fluids; (iii) concentrations of different markers detected in two wound fluids as measured by the hydrogel sensors or commercial assay kits [230]. (**b**) An injectable conductive Alg-PBA/PVA/GOH hydrogel with self-healing, motion monitoring, and bacteria theranostics for bioelectronic wound dressing: relative resistance changes when monitoring (i) human finger bending, (ii) index finger at different bending angles between 0° and 90°, (iii) human wrist bending, and (iv) human elbow bending [145]. (**c**) P(AM-APBA)XLG/CNTs hydrogel: (i), (iii) digital photographs and infrared thermal images of mouse skin incision after 3 and 7 days of healing, respectively; (ii), (iv) relative resistance changes of the P(AM3-APBA0.06)XLG1.0/CNTs hydrogel to detect the temperature of skin wounds on days 3 and 7, respectively; (v) relative changes of ΔR/R0 in wound detection on days 3 and 7, (vi) immunofluorescence images of CD11b (red), TNF-a (green), and nuclei (blue) in skin wound tissues (scale bar = 100 mm) [116].

Recent studies have reported the preparation of real-time monitoring carbon-based hydrogels for wound treatment and management. For example, Zheng et al. developed a CD-doped hydrogel sensor array in polydimethylsiloxane (PDMS) for simultaneous colorimetric detections of five wound biomarkers including pH, glucose, urea, uric acid, and total protein, holistically assessing inflammation and infection (Figure 9a) [230]. The sensor array exhibited high accuracy with recovery rates of 91.5–113.1% and clinically relevant detection ranges for all five wound markers, which was also validated with rat wound fluids from perturbed wound models and clearly distinguished wounds visually and quantitatively by distinct color patterns. Wang et al. designed a diagnostic and therapeutic hydrogel (LAMC/CD-C@M@P) to monitor the pH variation at wound sites and modulate the microenvironment of diabetic wounds through ROS scavenging and photothermal therapy [231]. By incorporating CDs, the hydrogel exhibited fluorescence responsiveness over a pH range from 4 to 9. And the fluorescence signals were detected using smartphones to measure the red, green, and blue (RGB) values, yielding a well-fitted linear curve. Therefore, dynamic pH values can be rapidly obtained, which can help reflect the wound condition. Besides, an integrated photo-inspired antibacterial PAI (PVA-iodine)/CMC/CQDs hydrogel dressing was reported for pH real-time monitoring and accelerated wound healing [232]. Due to the color responsiveness of PAI and CQDs to the pH of wound tissues under visible and UV light, a smartphone was utilized to collect and convert the pH sensing images to RGB signals to visually capture the real-time pH values during the wound healing process. Another pH-sensitive CDs/CS hydrogel with effective antibacterial properties could also be introduced as an excellent candidate for monitoring pH during the wound healing process [41].

Moreover, after elastic hydrogel substrates are doped with conductive inorganic nanomaterials or polymers, their conductive stability can be maintained by transmitting electrical signals through electrons and holes [233]. And with the addition of CBNs, flexible CBNHs can be employed as wearable electronic devices to monitor human motion [164,185,234,235]. Yan et al. synthesized conductive PGO-hybridized cellulose (PGC) bio-nanosheets and a PGC bio-nanosheet-assembled hydrogel (PGCNSH) with good mechanical flexibility, conductivity, and cell/tissue affinity, which could not only record electromyogram (EMG) signals and electrocardiogram (ECG) signals as outputs, but also served as an “E-skin” to efficiently transmit electrical stimulation to accelerate diabetic wound healing [183]. By incorporating the hydroxylated graphene (GOH) into the hydrogel networks, the self-healing injectable hydrogel (Alg-PBA/PVA/GOH) with in situ bacterial sensing and non-antibiotic killing properties displayed great electromechanical performance to achieve real-time monitoring and prevent re-tearing of the wound at human joints (Figure 9b) [145]. During the wound healing process, inflammation, vasodilation, immune responses and infection could all result in a temperature increase in skin wound tissue. Correspondingly, Shen et al. employed a P(AM-APBA)XLG/CNTs hydrogel for precisely monitoring large (e.g., elbow flexion, knee flexion, and running) and tiny movements (e.g., breathing, swallowing, and grabbing a cup) of humans. The multifunctional wearable hydrogel exhibited a tensile strength of 323 kPa, fracture strain of 1200%, compressive strength of 13.7 MPa, and fracture energy of 1078 J m^−2^. Notably, the detection of the ΔR/R0 based on the P(AM3-APBA0.06)XLG1.0/CNTs hydrogel reflected the temperature change due to its temperature sensing capability (Figure 9c) [116].

## 5. Conclusions and Future Perspectives

In this review, we overviewed the recent progress in the preparation and applications of multifunctional CBNHs. The low-dimensional CBNs, including CDs, GQDs, fullerenes, NDs, CNTs, graphene and its derivatives, are introduced into hydrogel networks via different interaction mechanisms, generating numerous CBNHs with a series of properties, such as self-healing, injectability, antibacterial, conductivity, anti-oxidative and anti-inflammatory performance, adhesion, hemostasis, substance release, and real-time monitoring. And we further summarized the functionalization of CBNs, including CDs, CNTs, and GO, and analyzed the effects on the properties of hydrogels resulting from the interactions between CBNs and other nanomaterials. In addition, multifunctional CBNHs can not only meet the functional requirements of different chronic wounds, such as infected, diabetic, and burn wounds, but also monitor body motion and wound parameters in real time, such as pH value, glucose level, and temperature. Hence, multifunctional CBNHs may play a significant role in the clinical treatment and management of chronic wounds.

Nevertheless, future research into CBNHs may be faced with several challenges. First, the cross-scale manufacturing method of CBNHs employing different CBNs of different dimensions is expected to be developed, and some properties are prospective, such as anti-cancer, anti-radiation, radiation refrigeration, etc. Second, the integration of various functions into one CBNH network is needed for meeting the diversity of wound treatment. Third, it is hard to achieve massive production of multifunctional CBNHs for biomedical or clinical applications.

## Figures and Tables

**Table 1:** Multifunctional hydrogels based on different functionalized GO nanomaterials.

Nanocomposite Hydrogels	Functionalization of GO	Interaction Mechanism	Functional Requirements	References
QNGH	quaternized N-halamine (QN)	Hydrogen bonds	Conductive, antibacterial, and real-time monitoring properties	[153]
PEI-rGO-PDA	polyethyleneimine (PEI)	Covalent bonds, hydrogen bonds	Self-adhesive, photothermal, and antibacterial properties, certain anti-inflammatory effect	[154]
ICG-PGO-CaP-PVA	PDA, indocyanine green (ICG)	Reversible hydrogen bonds	Photothermal and photodynamic antibacterial effects, self-healing, Ca^2+^ release, electroactivity, ROS-scavenging activity	[155]
CS-CGO	CS	Hydrogen bonds	High strength, excellent biocompatibility	[156]
rGO/MoS_2_/Ag_3_PO_4_	MoS_2_, Ag_3_PO_4_	Strong interfacial interactions	Photothermal and photodynamic antibacterial properties, certain anti-inflammatory effects, radical scavenging activity	[157]
PDA@Ag5GO1	PDA, Ag NPs	Covalent bonds	Self-adhesive and antibacterial properties, NIR-driven shrinkage, certain anti-inflammatory effects	[158]
rGB/QCS/PDA-PAM	3-Aminophenylboronic acid	Phenol–amine covalent bonds, dynamic borate ester bonds, hydrogen bonds, π–π stacking	Photothermal antibacterial activity, ROS production, adhesion, self-healing, hemostasis, and bacterial capture ability	[159]
QCS-CD-AD/GO	β-cyclodextrin	Host–guest interaction, hydrogen bonds	Injectable self-healing properties, conductivity, photothermal antibacterial activity, certain anti-inflammatory effects	[160]
rGO@PDA/Ag-PF127	PDA, Ag NPs	Dynamic borate ester bonds, thermo-reversible gel–sol transition	Photothermal–chemical antimicrobial performance with Ag^+^ release, adhesive, antioxidant, and hemostatic properties, certain anti-inflammatory effects	[161]
GO-BPEI/CMCS/PEG-CHO	Branched polyethyleneimine (BPEI)	Dynamic Schiff base bonds	Injectable, self-healing and photothermal properties	[162]
QCS/rGO-PDA/PNIPAm	PDA	Schiff base bonds, hydrogen bonds, cation–π interaction	Thermoresponsive self-contraction, tissue adhesion, temperature-dependent drug release, conductive, self-healing, antibacterial, antioxidant, and anti-inflammatory properties	[163]
Hep-PDA-rGO-PAM	Hep, PDA	Hydrophobic bonds, hydrogen bonds, ionic forces	Conductive, antibacterial, antioxidative, and real-time motion monitoring properties	[164]
β-GO/RB/PVA	-NH2, β-CD-DA	Hydrogen bonds	Photothermal and photodynamic antibacterial properties, certain anti-inflammatory effects	[165]
ZnO QDs@GO-CS	ZnO QDs	Electrostatic interactions	Photothermal and chemodynamic antibacterial activity with Zn^2+^ release and ROS generation	[166]
SF/CMC/AG&GO@PDA	PDA	Covalent bonds, hydrogen bonds	Injectable, conductive, antibacterial, hemostatic, and anti-inflammatory properties	[167]
Cu, N-CDs@GO-CS	Cu, N-CDs	Electrostatic interactions	Photothermal, photodynamic and inherent antibacterial effects	[67]
Gel/GO-βCD-BNN6	β-CD, BNN6	Covalent bonds, hydrogen bonds	Photothermal effect, NO release, antibacterial activity, anti-inflammatory effect, adhesiveness	[168]
GATP-PVA	Ag NPs, TGA	Hydrogen bonds, π–π interactions, electrostatic interactions	Electroactive, self-healing, tissue adhesive, antibacterial, and antioxidant properties, autolytic debridement	[169]
PC/GO/Met	PDA	Schiff-base bonds, phenylboronate ester dynamic bonds	pH/glucose dual-responsive metformin release, adhesive, self-healing, antibacterial, antioxidant, conductive, hemostatic, and anti-inflammatory properties	[170]
GelDA/pGO	PDA	Covalent bonds	Adhesive, hemostatic, conductive, antioxidant, and photothermal antibacterial properties	[171]
CMCS/THB/Cu/GB	BNN6	Dynamic Schiff base bonds, coordination complexation, non-covalent interactions	Conductive, self-healing, antioxidant, and photothermal antibacterial properties, NO release	[172]
GelMA/C-CNF/GelMA-DA	PDA	Covalent bonds	Adhesive, hemostatic, conductive, antioxidant, and photothermal antibacterial properties	[173]
ABA-GO/CNC/CMCS	3-Aminobenzene boronic acid	Electrostatic interaction, hydrogen bonds	Photothermal antibacterial, bacterial capture, and anti-inflammatory abilities	[174]
FC-rGO-PDA	PDA	Covalent bonds, π–π stacking, hydrogen bonds, electrostatic interactions	Antibacterial, hemostatic, and tissue adhesive properties	[175]
GO-arabinoxylan/PVA	Arabinoxylan	Covalent bonds, hydrogen bonds	Antibacterial and anticancer activities	[176]
PEP-AG	Ag NPs	Ag–amino coordination interaction	Thermoresponsive, sprayable, and antibacterial properties	[177]
OP/CMCS-RGO	CMCS	Schiff base condensation, hydrogen bonds	Self-healing and conductive properties, drug/photothermal antibacterial activity	[178]
GDFE	PDA	Hydrophilic-hydrophobic interaction, hydrogen bonds, Schiff base bonds	Injectable, thermosensitive, self-healing, antibacterial, antioxidant, conductive, and anti-inflammatory properties	[179]
IFI6-PDA@GO/SA	PDA	Hydrogen and π bonding interactions	Sprayable, antibacterial, and antioxidant characteristics	[180]
CGAPL	Ag NPs, ε-poly-L-lysine	Hydrogen bond, electrostatic, Schiff base, and hydrophobic/π–π interactions	Antimicrobial and anti-inflammatory effects	[181]
HA-DA/rGO	PDA	Covalent bonds, hydrogen bonds, π–π stacking	Adhesive, antioxidative, hemostatic, conductive, photothermal antibacterial, and drug release properties	[182]
PGCNSH	PDA	Covalent bonds, hydrogen bonding	Conductive and implantable properties, physiological signals detection	[183]
GO-f-BC/gelatin	bacterial cellulose (BC)	Covalent bonds, hydrogen bonding	Drug release, antibacterial activity	[184]
GOH-MPG	PDA, Fe_3_O_4_	Schiff base bonds, hydrogen bonds	Anisotropic, conductive, and photothermal antibacterial properties, rehabilitation training monitoring, certain anti-inflammatory effects	[185]
SDS-rGO-NaDC	Sodium dodecylsulfate	Hydrogen bonding, π–π stacking, hydrophobic interactions	Antibacterial activity	[186]
BCHA	Bi_2_S_3_, TiO_2_ NPs	Dynamic imine bonds, hydrogen bonds	Adhesive, photovoltaic, conductive, and anti-inflammatory properties, free radical scavenging ability	[187]
